# Succession of a natural desert vegetation community after long-term fencing at the edge of a desert oasis in northwest China

**DOI:** 10.3389/fpls.2023.1091446

**Published:** 2023-02-17

**Authors:** Yan Zhang, Guohua Wang, Qianqian Gou, Yu Zhang, Jing Liu, Min Gao

**Affiliations:** ^1^ College of Geographical Sciences, Shanxi Normal University, Taiyuan, China; ^2^ Key Laboratory of Desert and Desertification, Northwest Institute of Eco-Environment and Resources, Chinese Academy of Science, Lanzhou, China; ^3^ Linze Inland River Basin Comprehensive Research Station, Chinese Ecosystem Research Network, Northwest Institute of Ecology and Environmental Resources, Chinese Academy of Sciences, Lanzhou, China

**Keywords:** edge of desert oasis, plant diversity, natural fencing, nursing effect, soil physicochemical properties

## Abstract

Fencing is the most economical method of restoring degraded desert ecosystems, and plays an important role in promoting plant community diversity and productivity, as well as stable ecosystem structure and function. In this study, we selected a typical degraded desert plant community (*Reaumuria songorica*–*Nitraria tangutorum*) on the edge of a desert oasis in the Hexi Corridor in northwest China. We then investigated succession in this plant community and corresponding changes in soil physical and chemical characteristics over 10 years of fencing restoration to analyze the mutual feedback mechanisms. The results showed that: 1) The diversity of plant species in the community increased significantly over the study period, especially the number of herbaceous layer species, which increased from four in the early stage to seven in the late stage. The dominant species also changed, with the dominant shrub layer species shifting from *N. sphaerocarpa* in the early stage to *R. songarica* in the late stage. The dominant herbaceous layer species changed from the annual herb *Suaeda glauca* in the early stage to *S. glauca* and *Artemisia scoparia* in the middle stage, and ultimately to *A. scoparia* and *Halogeton arachnoideus* in the late stage. In the late stage, *Zygophyllum mucronatum*, *H. arachnoideus*, and *Eragrostis minor* began to invade, and the density of perennial herbs also increased significantly (from 0.01 m^-2^ to 0.17 m^-2^ for *Z. kansuense* in year seven). 2) As the duration of fencing increased, the soil organic matter (SOM) and total nitrogen (TN) contents first decreased then increased, whereas the available nitrogen, potassium, and phosphorus contents showed the opposite trend. 3) Changes in community diversity were mainly affected by the nursing effects of the shrub layer, as well as soil physical and chemical properties. That is, fencing significantly increased the vegetation density of the shrub layer, which promoted growth and development of the herbaceous layer. However, community species diversity was positively correlated with SOM and TN. The diversity of the shrub layer was positively correlated with the water content of deep soil, whereas that of the herbaceous layer was positively correlated with SOM, TN, and soil pH. The SOM content in the later stage of fencing was 1.1 times that in the early stage of fencing. Thus, fencing restored the density of the dominant shrub species and significantly increased species diversity, especially in the herb layer. Studying plant community succession and soil environmental factors under long-term fencing restoration is highly significant for understanding community vegetation restoration and ecological environment reconstruction at the edge of desert oases.

## 1 Introduction

Desert oases are core agricultural areas characterized as fertile and fragile ecosystems in arid or hyper-arid areas (Zhao et al., 2010; Chen et al., 2019; Chen et al., 2020). Oases are a unique geographic entity that appear as well-vegetated “islands” within drylands or deserts and can exhibit expansion (oasification) or retraction (desertification) under the combined effects of over-reclamation, overgrazing, and severe climate conditions (Wang, 2022). During the last 50 years, continuous development of oasis agricultural land has increased the area of oases in drylands worldwide, with the reclamation of large areas of natural shrubs and grasslands at the oasis edge leading to a decline of natural vegetation and desertification (Wang, 2009; Xu et al., 2019; Xue et al., 2019). In response to these environmental problems, various ecological projects have been implemented, with natural fencing and the artificial planting of sand-binding vegetation representing the main ecological restoration measures. In northwest China, such measures have effectively contributed to restoration of the local environment (Li et al., 2013).

Fencing is an important method of restoring and re-establishing arid desert areas characterized by less severe vegetation destruction and light wind-blown erosion. Research related to the fencing-based restoration of desert vegetation mainly deals with the characteristics of above-ground vegetation communities (Xu et al., 2015; Du and Gao, 2021), grassland diversity (Wang et al., 2012; Lu et al., 2021), plant biomass (Yang et al., 2005; Chen et al., 2006), herbal productivity (Yayneshet et al., 2008), community functional traits (Zhang et al., 2017), and dynamic changes of soil physicochemical properties (Du et al., 2007; Wang et al., 2012). Moreover, fencing can promote the succession of desert plant communities. Bruelheide et al. (2011) studied the potential plant community aggregation process reflected by the gradient of species composition in each successional stage, and identified clear successional stages of species composition. In addition, the soil environment has a significant influence on community succession (Zhang et al., 2011). For example, Li et al. (1999) studied vegetation succession in the Mu Us Desert area and noted that reasonable anthropocentric disturbance is required to prevent community degradation and ensure grassland quality during community succession. The soil nutrient status also directly affects the species diversity of plant communities. For example, Song et al. (2008) found that the Pielou index of species in Hunshandak Sandland was significantly correlated with soil pH, whereas the total nitrogen (TN) content was significantly correlated with the soil organic matter (SOM) content. Jia et al. (2021) investigated the diversity of pearl hogweed communities on the Alaska Plateau, and a significant positive correlation between the density of *Salsola passerina* communities and annual precipitation, soil water content, SOM, and TN content. Previous studies have predominantly involved short-term monitoring of desert plant communities; thus, continuous long-term analyses of the relationship between community changes and soil environmental factors are rare.

The edges of desert oases in the Hexi Corridor represent typical oasis-irrigated agricultural areas in the arid zone of northwestern China (Chang et al., 2012; Zhao et al., 2018). Here, grazing, as the main local human disturbance, is largely responsible for degradation and desertification of the grassland ecosystems (Gremer et al., 2015). In response, fencing management at the edges of the desert oases has been an important local ecological restoration measure for effectively promoting the structural recovery of degraded grassland ecosystems (Chen et al., 2022). In this study, we analyze the effects of long-term fencing on vegetation diversity and soil factors, as well as the relationship between plant community diversity and biotic and abiotic factors in the gravelly desert ecosystem of the Hexi Corridor. Specifically, we monitor continuous dynamic changes in the soil water content, soil nutrients, meteorology, and desert plant communities during the last 10 years, and provide a reference for the restoration and ecological protection of the gravelly desert ecosystem of the Hexi Corridor. We hypothesize that, over time, fencing restoration leads to 1) a progressive increase in plant species richness with ecological succession; 2) improved soil nutrient contents as the plant communities change, facilitating the colonization and establishment of additional species.

## 2 Materials and methods

### 2.1 Study site

**Figure 1:**
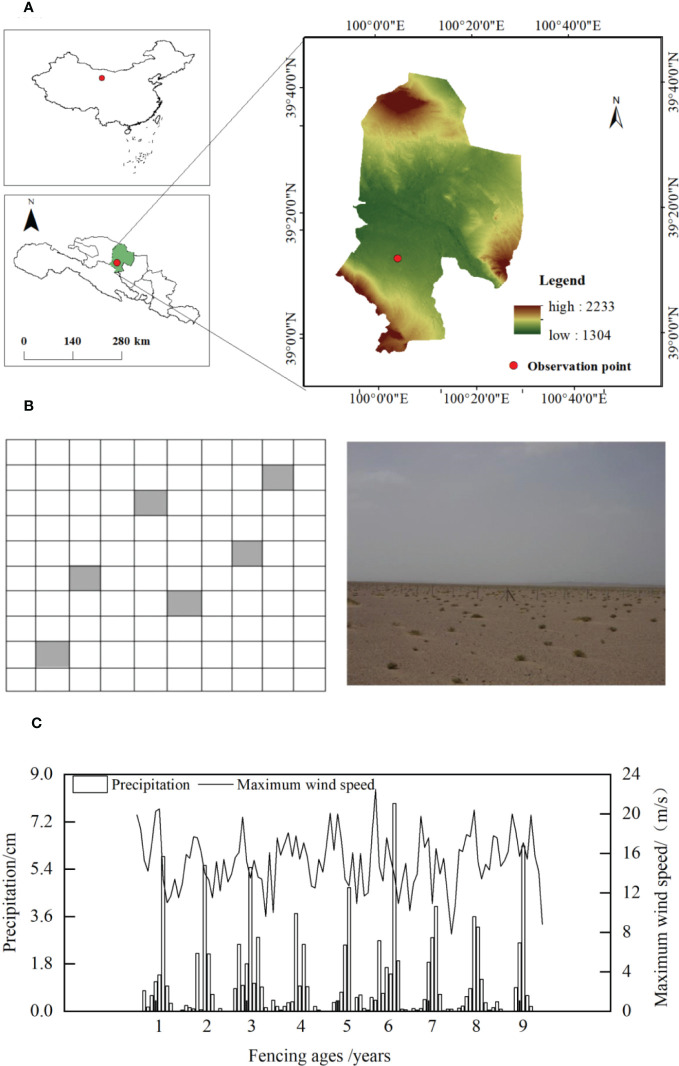
Location of the study area. **(A)** Location of the study area; **(B)** Study area soil sample plot setup; **(C)** Dynamic of precipitation and maximum wind speed in the study area during the fencing period.

The study area was located at the Linze Inland River Basin Integrated Research Station (LIRBIRS, 39° 21’ N, 100° 07’ E, 1,367 m above sea level) of the Ecosystem Research Network of the Chinese Academy of Sciences, in Pingchuan Town, Linze County, in the central part of the Hexi Corridor, Gansu Province, China (**Figure 1A**). The study area has a temperate continental desert climate, characterized by sparse precipitation throughout the year, which is concentrated in summer, as well as a dry climate, long day lengths, and intense solar radiation; the annual precipitation is 124.3 mm and the average annual temperature is 7.6°C. The annual average wind speed is 3.2 m·s^-1^, the maximum wind speed is 21 m·s^-1^, and the prevailing wind direction is northwesterly (Zhao et al., 2003). The precipitation and maximum wind speed dynamics during the fencing period (**Figure 1C**), which were determined using meteorological data obtained from the desert ecosystem meteorological long-term experiment sample sites at LIRBIRS, indicated fluctuations but no significant annual increases or decreases. The study area is dominated by semi-fixed dunes with a simple plant community structure and few species. Xerophytes and semi-shrubby vegetation dominate. The xerophytes and ultra-xerophytes do not typically include short-lived or annual plants. Representative plants include *Reaumuria songorica*, *Nitraria tangutorum*, *Calligonum chinense*, *Haloxylon ammodendron*, and *Agriophyllum squarrosum*, which exhibit a mixed spatial distribution, constituting a unique patch vegetation pattern. The dominant herb species are *Artemisia scoparia, Suaeda glauca, Halogeton arachnoideus, and Eragrostis minor*, with sporadic *Zygophyllum kansuense* and *Allium mongolicum*.

### 2.2 Sample site setup and survey

#### 2.2.1 Sample plots for soil observations during the fencing period

The soils and plants at the Integrated Observation Site for Sequestered Desert Ecosystems, LIRBIRS, were continuously monitored for almost 10 years. The dominant fencing measure was barbed wire fencing, which was employed to reduce disturbance by human-mediated grazing activities. Six 10 × 10 m sample plots were set up diagonally across the site for soil sampling, with each plot separated by more than 20 m (**Figure 1B**). Three neutron tubes were set up evenly along the diagonal of the site to measure the soil water content. The soil particle composition and bulk density were measured every five years from the first year of fencing (**Table 1**).

**Table 1:** Changes of soil particle composition and soil bulk density during fencing.

Fencing ages		1		6	
		0-10cm	10-20cm	0-10cm	10-20cm
soil particle composition	Sand%	86.06	84.22	74.46	73.68
	Silt%	11.26	12.78	21.74	22.44
	Clay%	2.68	3.00	3.80	3.88
soil bulk density	g/cm^3^	1.67	1.63	1.68	1.6

#### 2.2.2 Soil nutrient analysis

Soil samples were obtained by soil auger. A 5-cm diameter soil auger was used to drill along the diagonal (two corners and the center) of each plot to obtain soil at a depth of 0–20 cm from the surface. Soil samples were then transported to the laboratory to analyze the soil physicochemical properties. SOM content was determined by the potassium dichromate oxidation method; total phosphorus was determined by the sulfuric acid – perchloric acid cooking – molybdenum antimony anti-colorimetric method; available phosphorus was determined by the sodium bicarbonate leaching – molybdenum antimony anti-colorimetric method; fast-acting potassium by the ammonium acetate leaching – flame photometric method; fast-acting nitrogen was determined by the alkali diffusion method; pH was determined using a water–soil ratio of 2.5:1.0 and the potentiometric method; and soil water content was determined using a neutron meter. The soil water content depth was 0–150 cm. We used the soil water content at 0–40 cm as the shallow soil water content, that at 40–90 cm as the middle soil water content, and that at 90–150 cm as the deep soil water content.

#### 2.2.3 Sample plots for vegetation observations during the fencing period

During the 10 years of fencing, we investigated the vegetation community in the sample plots in August each year; non-fenced quadrats were selected as a blank control (ck). A transect measuring 100 m in length and 10 m in width was set along the observation field, with a total of 10 quadrats of 10 × 10 m. Because of the small number of plants, the sample square was divided into four equal parts with colored fiber ropes, and the number of each plant species was counted. Ten plants were randomly selected from the sample square, and their mean height was calculated (plant heights were measured using steel tape). If there were fewer than 10 plants present, the height of all plants was measured, and the mean value was calculated from the available data. Thus, we determined the species, number, height, and growth form of the plants in each sample plot.

### 2.3 Community analysis and statistics

The importance value, Margalef richness index (*M*a), Shannon–Wiener diversity index (*H*), and Pielou evenness index (*E*) were selected to express the *Reaumuria songorica–Nitraria tangutorum* community diversity through a statistical analysis of the community quantitative characteristics using the following formulas:


(1) IVs=(Relative density+Relative height+Relative frequency)/3

where the relative density is the density of a species/sum of the densities of all species; the relative height is the plant height of a species/sum of plant height of all species; the frequency of species i in the sample was first calculated as the ratio of the number of species i in the sample to the total number of species in the sample. then, the frequencies of all species in the sample square were calculated.

Margalef richness index:


(2) Ma=(S−1)/lnN

where S is the number of species in the sample square; and N is the number of all plants in the sample square.

Shannon–Wiener diversity index:


(3) $$H=-\sum_{i=1}^{s}P_{i}InP_{i}$$

where P_i_ is the ratio of the number of individuals, n_i_, of species i in the sample to the total number of individuals, n, of the species in the sample, P_i_ = n_i/_n and i=1, 2, 3,…

Pielou index:


(4) E=H/Ln(S)

The data were prepared and statistically analyzed using Microsoft Excel and SPSS19.0 software. The significance of the difference between each soil nutrient parameter in different years of fencing and between different soil layers was tested by one-way ANOVA. Redundancy analysis (RDA) was conducted using Canoco 4.5 software to study the relationship between plant community diversity and environmental factors such as soil nutrients and water content. The correlations between biomass characteristics of the shrub and herb layers, and between species diversity and density, were analyzed using the performance analytics and GGally package in R R Core Development Team 2021 and plotted using Origin 2018.

## 3 Results

### 3.1 Changes in species composition and dominant species of plant communities after fencing

The number of species, height, and vegetation density in the plant community tended to increase with the number of years of fencing, and the community gradually evolved from simple to complex (**Table 2**). The plant community was divided into two different levels according to the growth form: a shrub layer and a herbaceous layer. The community had a total of six families, nine genera, and nine species, with two families, two genera, and two species of shrubs (22.2% of the total species richness), and five families, seven genera, and seven species of herbaceous plants (77.8% of the total species richness). Zygophyllaceae play an important role in the vegetation species, accounting for 33.3% of all species.

Prior to fencing, the shrub layer was dominated by *N. tangutorum*, with *R. songorica* becoming the dominant species from the second year of fencing and the herbaceous layer becoming dominated by *S. glauca* in the early stage of fencing (densities of 0.11 m^-2^ and 0.13 m^-2^, respectively). The dominant species in the middle stage of fencing (4–6 years) was *A. scoparia* (density = 0.24 m^-2^ in year six), whereas the dominant species in the late stage of fencing (7–9 years) were *A. scoparia* and *H. arachnoideus* (density = 0.43 m^-2^ in year seven). The density of *H. arachnoideus* increased to 1.48 m^-2^ in the ninth year of fencing; however, the perennial herb *Z. mucronatum* and the grass *E. minor* colonized at this stage, with the density of the latter reaching 0.83 m^-2^. Moreover, the density of the perennial herb *Z. kansuense* increased to 0.17 m^-2^ at this stage, and the plant community gradually stabilized (**Table 2**).

**Table 2:** Importance value of plant community after different fencing years.

Species	Type	ck			early stage									middle stage									late stage								
					1			2			3			4			5			6			7			8			9		
		h	D	IV_S_	h	D	IV_S_	h	D	IV_S_	h	D	IV_S_	h	D	IV_S_	h	D	IV_S_	h	D	IV_S_	h	D	IV_S_	h	D	IV_S_	h	D	IV_S_
Nitraria sphaerocarpa	S	21.3	0.056	89.9	12.6	0.04	64.9	15.3	0.08	19.2	27.7	0.06	24.2	28.2	0.10	30.5	24.6	0.09	33.5	18.1	0.13	32.2	26.7	0.11	17.5	22.5	0.09	16.3	27.5	0.12	15.2
Reaumuria songarica	S	18.25	0.018	10.1	20.5	0.02	52.0	14.7	0.17	29.3	20.3	0.15	39.0	20.7	0.25	51.5	20.6	0.25	58.9	13.6	0.16	36.1	20.6	0.41	29.3	19.0	0.47	40.0	21.7	0.45	21.6
Zygophyllum kansuense	P		—			—		3.4	0.05	8.5	4.3	0.02	7.3		—		1.8	0.02	5.6	3.8	0.01	5.1	2.6	0.17	8.9		—		1.5	0.01	0.7
Allium mongolicum	P		—			—		8.5	0.04	9.3	16.3	0.04	15.1	9.5	0.03	10.1	12.1	0.05	15.4		—		11.6	0.05	8.1	11.6	0.04	8.0	16.8	0.04	8.1
Zygophyllum mucronatum	P		—			—			—			—			—			—			—			—		2.8	0.02	2.4		—	
Suaeda glauca	A		—			—		3.7	0.11	17.1	9.4	0.13	26.6	23.9	0.18	27.4		—			—			—		7.3	0.03	4.6	5.0	0.03	3.6
Halogeton arachnoideus	A		—			—			—			—			—			—			—		4.8	0.21	11.9	7.0	0.15	14.2	7.8	1.48	35.6
Artemisia scoparia	A		—			—		15.3	0.12	22.5	22.4	0.05	17.5		—		15.2	0.08	17.7	12.4	0.24	34.6	15.8	0.43	27.6	13.7	0.23	20.2	15.5	0.01	5.7
Eragrostis minor	A		—			—			—			—			—			—			—			—			—		6.7	0.83	21.0
Community density			0.074		0.06			0.57			0.45			0.56			0.49			0.54			1.38			1.03			2.97		
Shrub layer density			0.074		0.06			0.25			0.21			0.35			0.34			0.29			0.52			0.56			0.57		
Herbaceous layer density			–		–			0.32			0.24			0.21			0.15			0.25			0.86			0.47			1.4		
Shrub layer species richness			2		2			2			2			2			2			2			2			2			2		
Herbaceous layer species richness			–		–			4			4			3			4			3			4			6			6		
Dominant species in the shrub layer		Nitraria sphaerocarpa			Nitraria sphaerocarpa			Reaumuria songarica			Reaumuria songarica			Reaumuria songarica			Reaumuria songarica			Reaumuria songarica-Nitraria sphaerocarpa			Reaumuria songarica			Reaumuria songarica			Reaumuria songarica		
Dominant species in the herbaceous layer		–			–			Artemisia scoparia			Suaeda glauca			Suaeda glauca			Artemisia scoparia			Artemisia scoparia			Artemisia scoparia			Artemisia scoparia			Halogeton arachnoideus		

A, Annual; P, Perennial; S, Semi − shrub;” − “ Represents that there is no this species. IVs/%, the importance value; h/cm, height; D, density/n/m^2^.

The community density increased significantly over the study period, and reached a maximum value of 40.14 times the initial community density in the ninth year of fencing. For example, the density of the shrub layer increased from 0.07 to 0.57, and the density of the herbaceous layer increased from 0.32 to 1.40. Notably, the density of *H. arachnoideus* increased from 0.21 to 1.48 in the late period (**Table 2**). Development of the vegetation community was characterized by the successful invasion of annual and perennial herbs and a gradual increase in the number of species.

### 3.2 Changes in plant diversity during the study period

**Figure 2:**
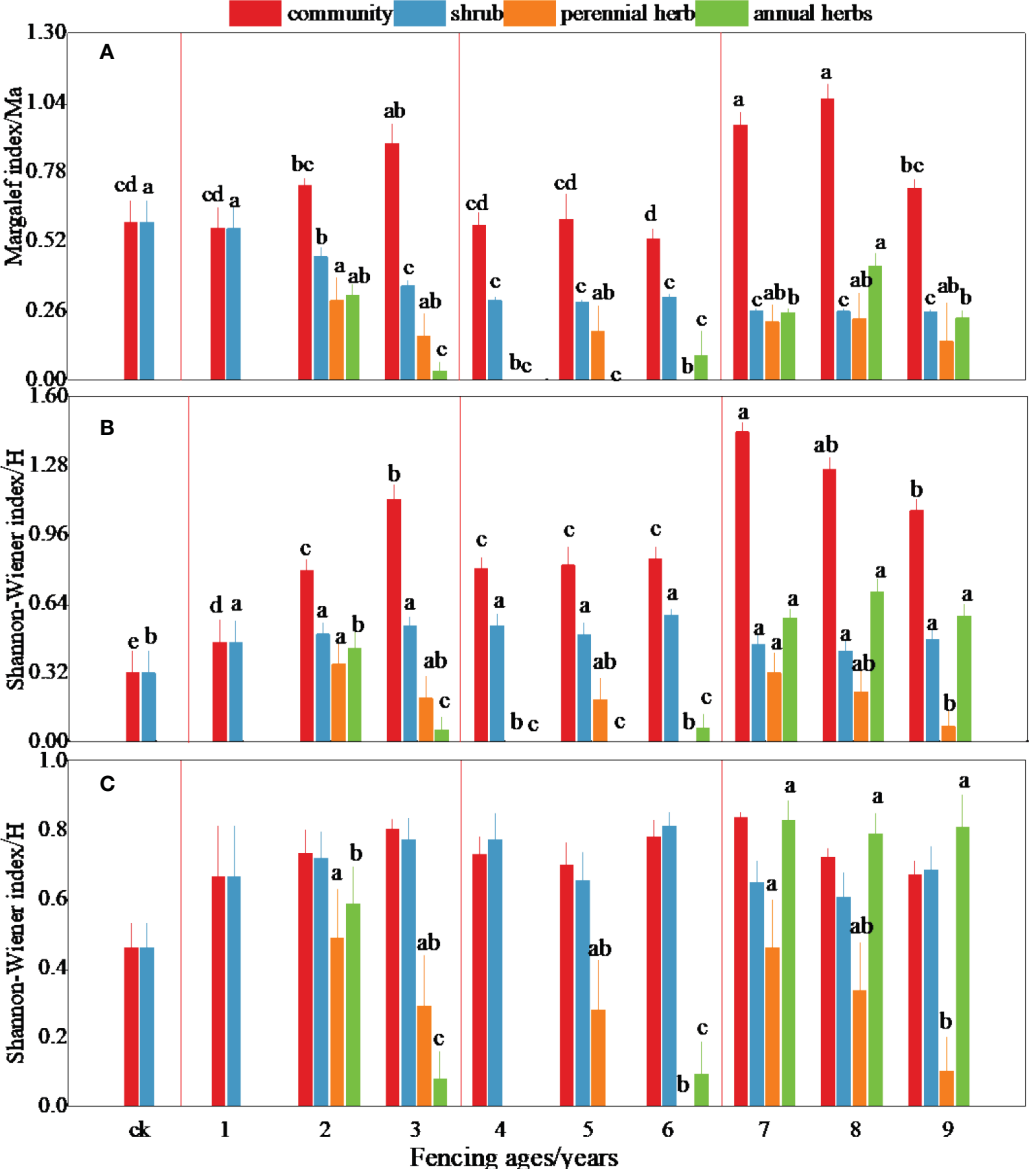
Dynamic changes of plant diversity in different fencing years. Different letters represent significant difference between different ages.

The species composition within the *Reaumuria songorica*–*Nitraria tangutorum* community was simple, with plant diversity from the early–middle–late stages of fencing first increasing, then decreasing, then increasing again (**Figure 2**). The community richness and diversity showed a significant increasing trend in the early stage (**Figures 2A–C**); the richness and diversity in the third year of fencing were 1.56 and 2.44 times higher than the initial values. The diversity index showed a significant decreasing trend, with the lowest value in the middle stage. The richness and diversity indices showed a significant increasing trend in the later stage, and the diversity of perennial as well as annual herb layer plants also increased significantly, causing the plant community structure to gradually stabilize.

### 3.3 Dynamic changes in soil water content and soil nutrients during the study period

**Figure 3:**
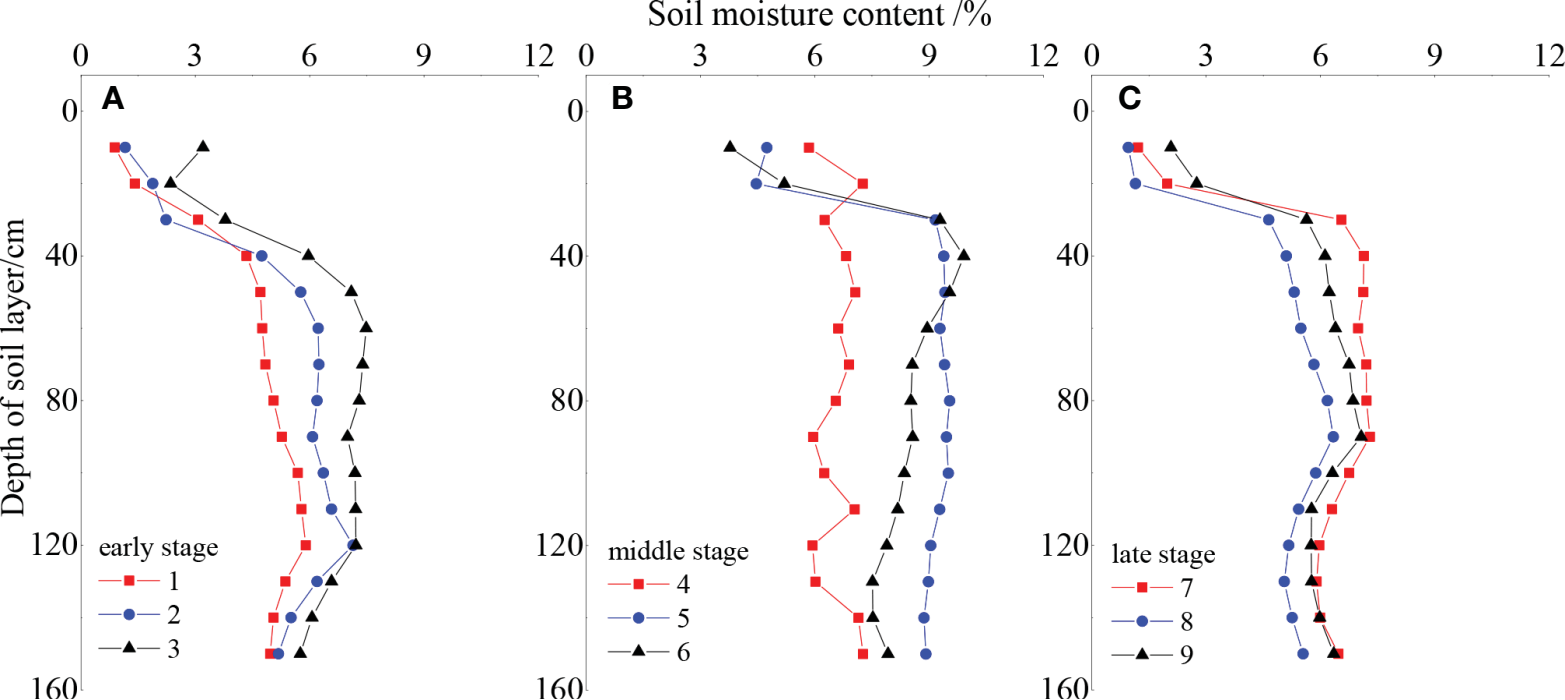
Dynamic of soil water content in different fencing periods **(A)** early, **(B)** middle and **(C)** late.

The soil water content of the *Reaumuria songorica*–*Nitraria tangutorum* community increased then decreased over time (**Figure 3**). Soil water content increased from 5.25% to 7.74% in the middle of the fencing period, and reached a maximum in the fifth year of fencing; it then decreased from 7.74% to 5.54% in the later years. The soil water content also increased then decreased with increasing depth of the soil layer. In the 0–40 cm soil layer, the soil water content increased significantly with depth; in the middle layer (40–100 cm), the water content was higher than that in the other layers; and in the deep layer (90–150 cm), the soil water content gradually stabilized. In general, the soil water content of the community increased slowly during the study period; however, an abnormally high value appeared in the 5th and 6th year of fencing because of high precipitation in these years. The soil water content then gradually stabilized in the late period, but remained higher than that in the early fencing period.

**Figure 4:**
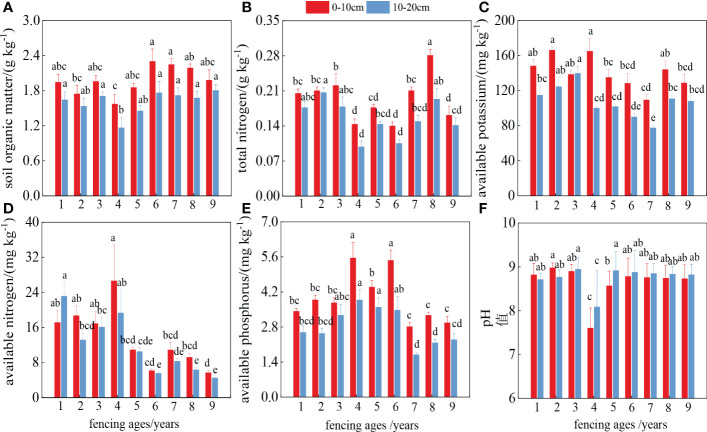
Dynamic changes of soil nutrients in different years. Different letters represent significant difference between different ages.

SOM and TN contents decreased in the middle period and recovered in the later period (**Figures 4A–C**). The available phosphorus and available nitrogen contents reached maximum values in the middle of the fencing period, and were 1.87 and 4.70 times higher in the fourth year than in the ninth year of fencing, showing a trend of increasing then decreasing, before finally reaching a stable state (**Figures 4D, E**). The pH value first increased then decreased over the study period (**Figure 4F**), falling to a minimum value of 7.61 in the fourth year of fencing before gradually stabilizing in the later years of fencing. Furthermore, the nutrient content in the shallow (0–10 cm) soil was higher than that at 10–20 cm, showing clear “surface aggregation.”

### 3.4 RDA analysis of plant communities and environmental factors

According to **Table 3**, the Eigen values of the first two axes of the community were 0.745 and 0.163, explaining 74.5% and 16.3% of the species-environment relationships, respectively, with a cumulative variance that explained 90.8%. Moreover, the Eigen values of 0.823 and 0.115 for the first two axes of the shrub layer explained 82.3% and 11.5% of the species-environment relationships, respectively, with a cumulative variance that explained 93.8%. The Eigen values of 0.952 and 0.046 for the first two axes of the perennial herbaceous layer explained 95.2% and 4.6% of the species-environment relationships, respectively, with a cumulative variance that explained 99.9%. Lastly, the Eigen values of the first two axes of the annual herbaceous layer were 0.811 and 0.153, which explained 81.1% and 15.3% of the species-environment relationships, respectively, with a cumulative variance that explained 96.4%. This reflects the ranking information more completely, indicating that the selected environmental factors can better reflect the distribution pattern of plant communities, and that the ranking results are credible.

**Table 3:** Results of RDA correspondence analysis between community and environmental factors of *Reaumuria soongoric* and *Nitraria tangutorum*.

	Statistical axis	Eigenvalue	Cumulative variation rate/%	
			Species data Variance	Species - Environment Relationships
Community	Axis 1	0.745	74.5	74.5
	Axis 2	0.163	90.8	90.8
	Axis 3	0.082	98.9	98.9
	Axis 4	0.010	99.9	99.9
Shrub layer	Axis 1	0.823	82.3	82.3
	Axis 2	0.115	93.8	93.8
	Axis 3	0.056	99.4	99.4
	Axis 4	0.006	100.0	100.0
Perennial herbaceous layer	Axis 1	0.952	95.2	95.2
	Axis 2	0.046	99.9	99.9
	Axis 3	0.001	100.0	100.0
	Axis 4	0.000	100.0	100.0
Annual herbaceous layer	Axis 1	0.811	81.1	81.1
	Axis 2	0.153	96.4	96.4
	Axis 3	0.034	99.9	99.9
	Axis 4	0.001	100.0	100.0

**Figure 5:**
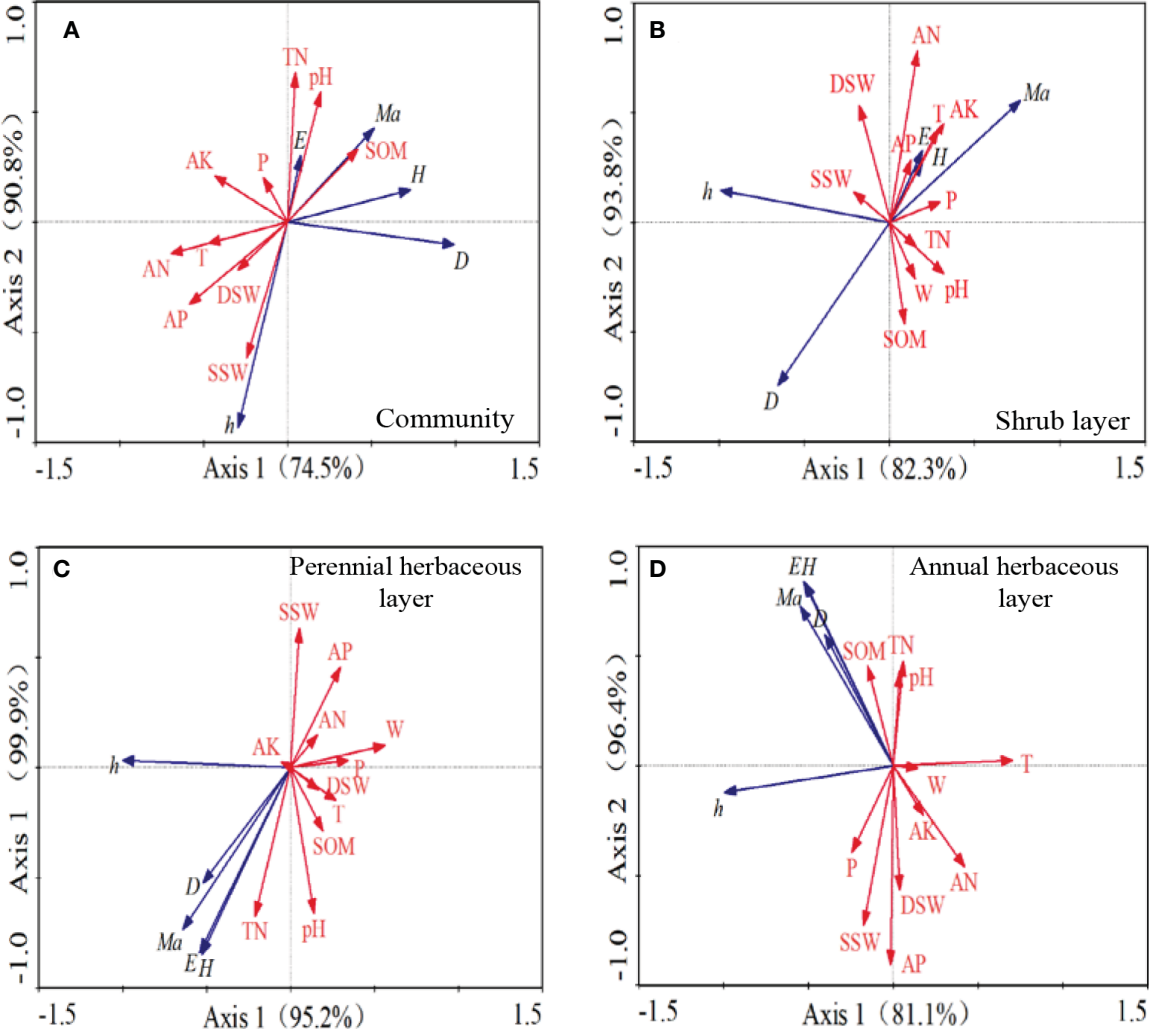
RDA constrained ordination analysis diagram of soil environmental factors, meteorological factors and plant community. AK, soil fast-acting potassium; AN, soil fast-acting nitrogen; AP, soil effective phosphorus; SOM, soil organic matter; DSW, deep Soil water content; SSW, shallow soil water content; pH, pH; D, density; h, height; H, Shannon-Wiener index; E, Pielou index; *Ma,* Margalef index; T, average growing season temperature; W, maximum growing season wind speed; P, growing season precipitation.

We observed significant correlations between different plant groups and environmental factors (**Figure 5**), with the correlation coefficients between different plant groups and soil and environmental factors equal to one. The Eigen values of axis 1 ranged from 74.5% to 95.2%, and those of axis 2 ranged from 4.6% to 16.3%. For the plant community (**Figure 5A**), the diversity, richness, and evenness indices were all affected by soil TN, SOM, available potassium, and pH, which exhibited positive correlations, and the community density was positively correlated with SOM. For the shrub layer (**Figure 5B**), the diversity and evenness indices were positively correlated with soil nutrients, such as available nitrogen and available potassium, soil pH, and deep soil water content, and both indices were positively correlated with the plant growing season temperature and precipitation. In terms of the perennial herbaceous layer (**Figure 5C**), the diversity and evenness index were positively correlated by soil pH, TN, and SOM, but negatively correlated with maximum wind speed during the growing season. As for the annual herbaceous layer (**Figure 5D**), the density, diversity, richness, and evenness indices were positively correlated with SOM, TN, and pH. The growth of annual herbs, as recorded by plant height, was greatly influenced by the shallow soil water content, whereas the diversity and uniformity of annual herbs were more influenced by the soil nutrients.

### 3.5 Shrub nursing effect

**Figure 6:**
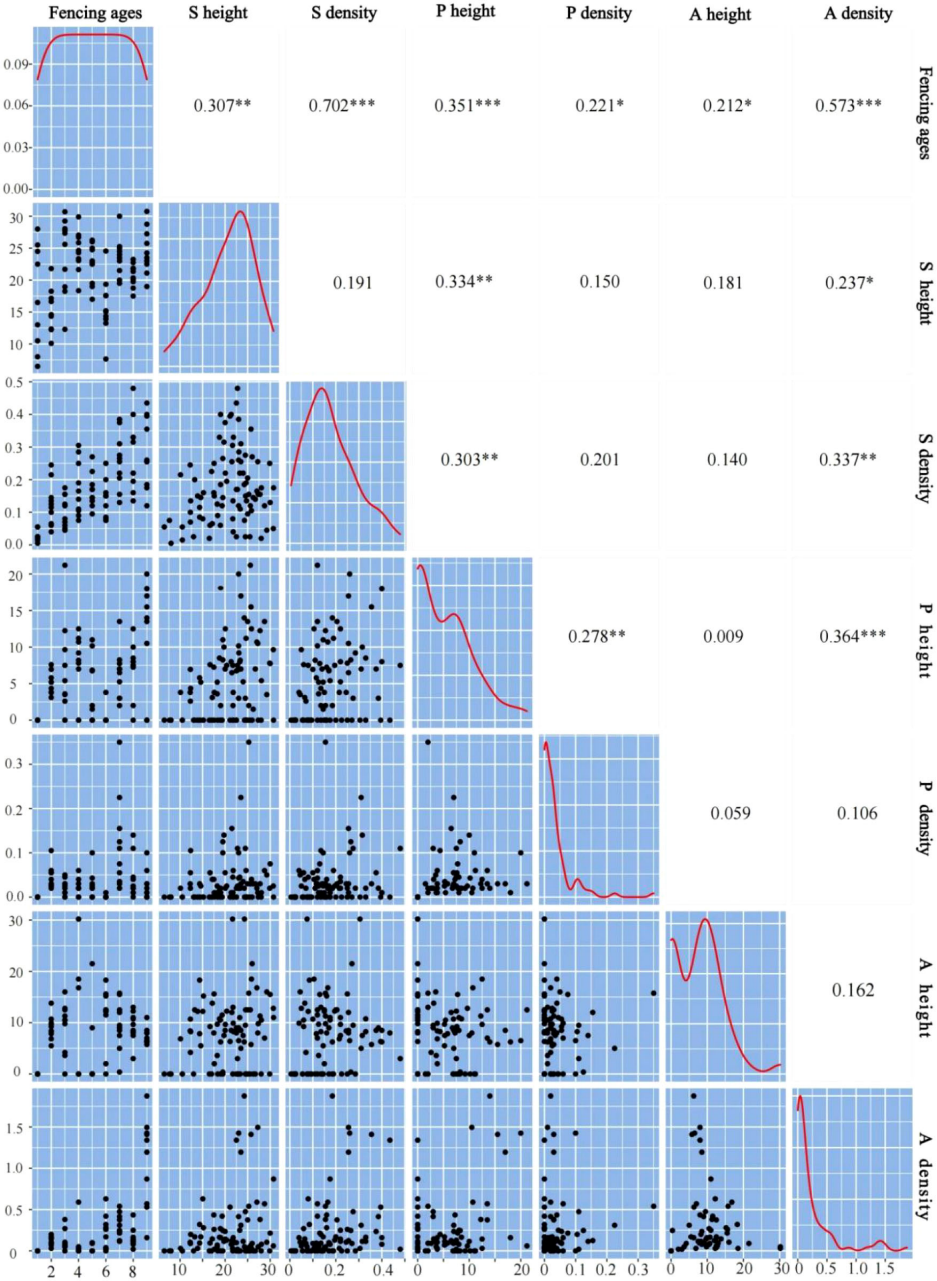
Correlation of biological indexes between shrub layer and herb layer. S height represent the shrub height; S density represent the shrub density; P height represent the perennial herb height; P density represent the perennial density; A height represent the annual herb height; A density represent the annual herb density. *p < 0.05; **p < 0.01; ***p < 0.001.

The duration of fencing and the plant community characteristics (height and density) of the shrub and herbaceous layers showed a significant positive correlation (**Figure 6**). Shrub height was significantly and positively correlated with perennial herb height and annual herb density, and the shrub layer density was positively correlated with perennial herb height and annual herb density. The increase of shrub layer plant density also had a promotional effect on perennial and annual herbs. When shrubs promote plant growth and settlement, or increase the species richness and diversity of herbaceous plants, a nursing effect is observed between species (Cahill, 2008). This nursing effect of shrubs directly promotes growth of the herbaceous layer, and occurs frequently in arid environments, which is an important reason for plant diversity.

### 3.6 Relationship between plant community species diversity and community density

**Figure 7:**
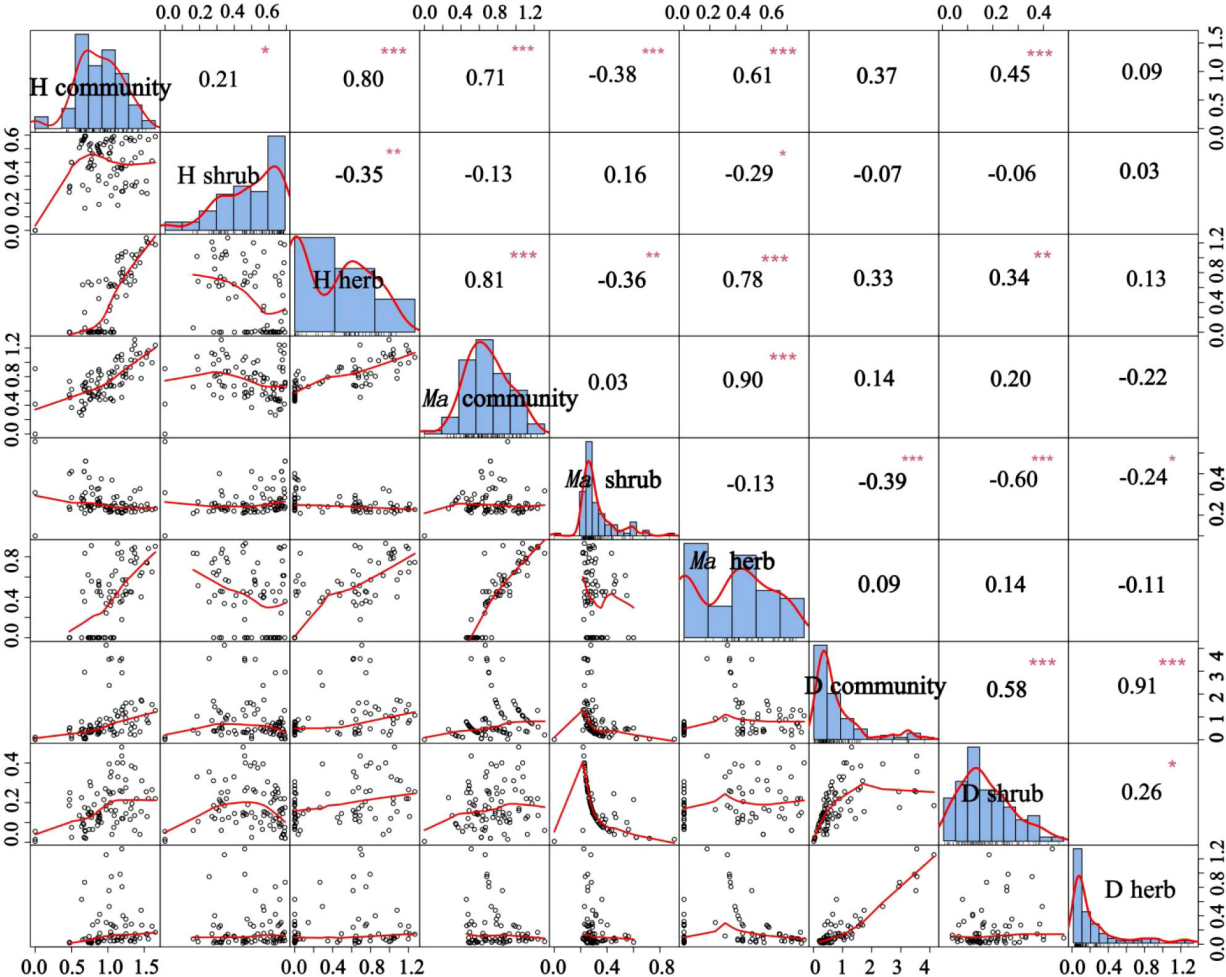
Relationship between community richness diversity and community density. H community, H shrub, and H herb represent the Shannon-Wiener diversity index of community, shrub layer, and herb layer; *Ma* community, *Ma* shrub, and *Ma* herb represent the Margalef richness index of community, shrub layer, and herb layer; D community, D shrub, and D herb represent the density of community, shrub layer, and herb layer. *P < 0.05, **P < 0.01, ***P < 0.001.

We observed a positive correlation between plant density and the number of species (**Figure 7**). This is because greater vegetation density changes the habitat conditions, resulting in an increase of surface roughness, which reflects the influence of vegetation density on soil wind erosion, and leads to an improved soil environment, the survival and growth of more plant seeds, and an increase of plant diversity (Dong et al., 1996).

## 4 Discussion

### 4.1 Effects of long-term fencing on the desert vegetation community

Most of the species in the studied community belonged to the family *Zygophyllaceae*, with relatively few other plant families present. This is typical of arid desert areas, where shrubs and semi-shrubs dominate and herbs mostly exist as companion species. This is attributed to the dry climate and strong wind erosion in arid desert ecosystems, where shrubs and semi-shrubs can better adapt to the stress of abiotic environmental factors (He and Zhao, 2004). Moreover, *R. songorica* replaced *N. tangutorum* as the dominant species in the shrub layer after fencing. This is mainly because *R. songorica*, as a medium forage grass with better palatability, is more susceptible to human grazing or wildlife activities than *N. tangutorum*, which is easily eaten by sheep and camels in the marginal areas of desert oases where plant species are very scarce. Therefore, *R. songorica* gradually recovered and eventually became the dominant species in the shrub layer after natural fencing and elimination of grazing and wildlife disturbance. The results of this study showed that the implementation of fencing measures can significantly promote the recovery of desert vegetation diversity. In the later stage of fencing, the species richness increased significantly, and Z. mucronatum, H. arachnoideus and E. minor began to colonize plots, which inevitably affected the growth of existing dominant herbs. As a result, the importance value of the previously dominant species, A. scoparia, decreased. However, from the perspective of colonizing species, annual herbs still dominated, which indicates that the recovery of desert plant communities is a slow and gradual process, and community succession in the enclosed study area was still in the transitional stage at the end of the study period.

In this study, the species diversity index was approximately consistent with the species richness, that is, the higher the number of species, the higher the diversity index, which is consistent with other studies in the Hunshandak sandy area (Qi et al., 2021) and western Inner Mongolia (Yang et al., 2017). Species diversity is characterized by both richness and evenness, and commonly indicated by the Margalef richness index and Shannon–Wiener diversity index (Jiang et al., 2022). The Pielou evenness index of plant communities was generally high in the study area, which is conducive to maintaining the stability of the community structure.

The results of this study indicated that fencing promoted an increase in shrub density, and that shrubs had a conservation effect on herbs. This is attributed to the fact that very few pioneer herbs can survive and reproduce in desert ecosystems, which are subject to wind damage, sand damage, and erosion, However, because of the ability of scrub to reduce the wind speed, intercept wind and sand, and improve the local microclimate (Zhao et al., 2006; Zhao et al., 2007), these plants promote the survival and development of herbaceous plants beneath, allowing some non-pioneer herbaceous plants to grow and reproduce normally (Francisco and Robert, 2000). Moreover, the species richness of herbaceous layer communities in desert ecosystems is generally higher than that of shrub layer communities, and competition among species of similar taxa for the same resources is intense, which leads to more obvious heterogeneity in the phylogenetic structure and successional development of herbaceous layer species (Chai et al., 2019).

### 4.2 Effects of soil factors on plant species diversity

Soil moisture and nutrients play an extremely important role in the degree of vegetation restoration, which is an important indicator of soil quality and soil health (Cai et al., 2020; Liu et al., 2022). In this study, we found that the moisture content of soil in the surface layer (0–40 cm) was low, that in the middle layer (40–120 cm) varied significantly but was significantly higher than that in the surface layer, and that in the deep layer (120–150 cm) was minimal. This is because the surface layer of the desert ecosystem is dominated by sand grains and has a weak water-holding capacity, whereas the middle layer is deposited with powder and clay grains, and exhibits greater soil water-holding capacity. In contrast, the transpiration rate decreases with increasing soil depth, so the soil water content increases to become the main source of soil water for plants (Lu et al., 2021).

In this study, soil nutrients continued to aggregate in the surface layer over the study period; however, the aggregation effect decreased significantly with increasing soil depth. This surface aggregation occurred because of restoration of the shrub- and herbaceous-layer vegetation after fencing, which protected the sub-surface from wind erosion (Wang et al., 2022). Furthermore, the deposition of fine particulate matter under the vegetation canopy, a process that proceeds continuously, leads to an increase in clay-powder particles in the soil surface layer and the accumulation of soil nutrients, especially SOM and TN. However, the available soil nutrient contents showed different trends. This is because the chemical form of fast-acting nutrients is water-soluble and easily mobile (Du et al., 2007). As the vegetation recovered, the plant density and cover increased each year, which would have greatly increased the consumption of available nutrients, resulting in lower levels.

**Figure 8:**
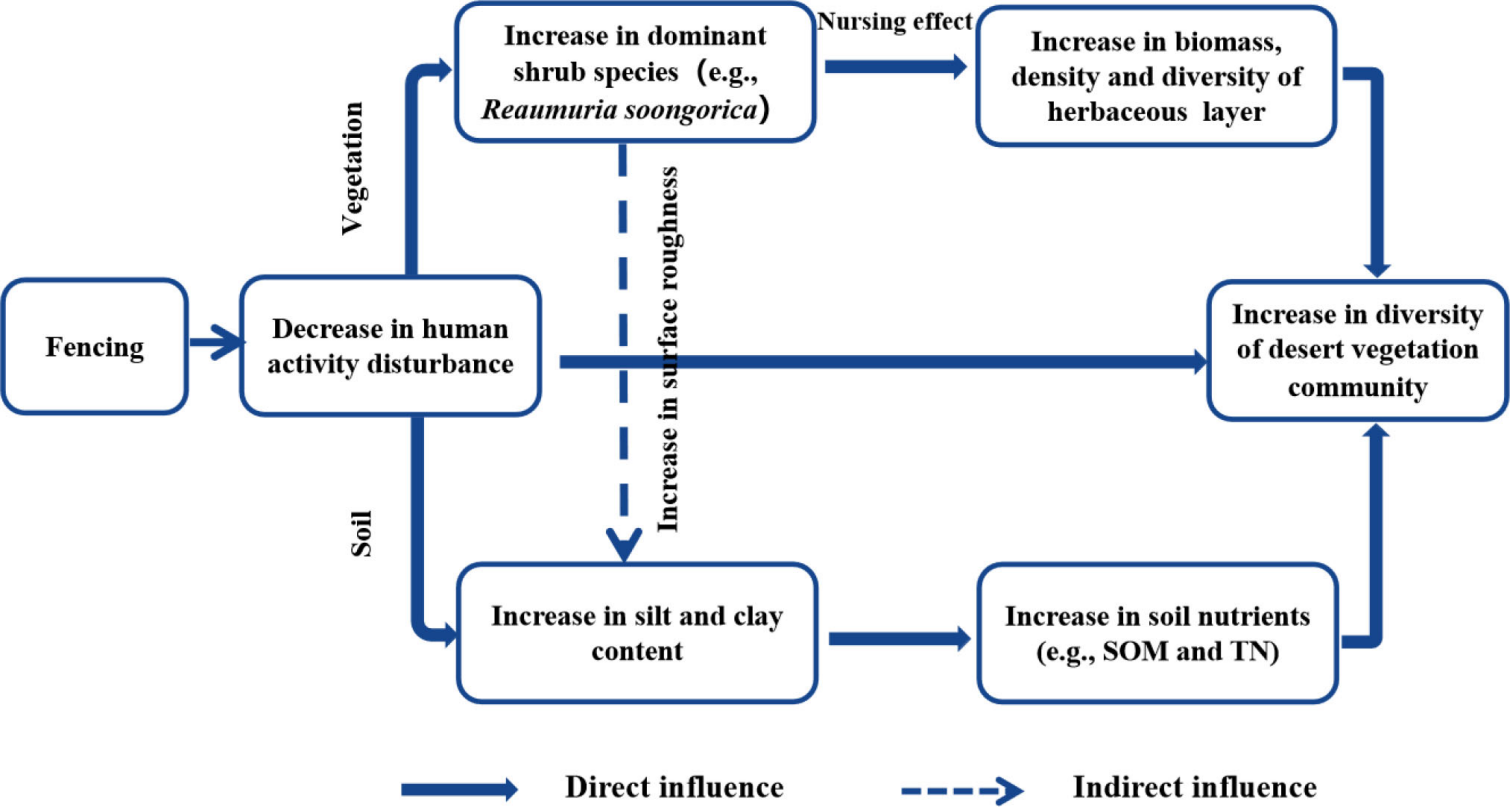
The conceptual framework of changes in desert vegetation diversity after fencing.

Species diversity is a key indicator of the number, distribution, and stability of species in a community or ecosystem, and includes the species richness, evenness, dominance, etc. (Carvalho et al., 2020). In this study, we found that both the diversity and density of perennial and annual herbaceous plants were influenced by soil SOM, TN, and pH. The soil nutrient status improved as the duration of fencing increased, which led to an increase of SOM and TN contents, providing conditions more conducive to plant survival. Thus, it is beneficial to promote the survival of surface herbaceous plants as they are important for the recovery of species diversity in arid zone desert ecosystems (Abramsky and Rosenzweig, 1984). We also observed a significant positive correlation between shrub layer diversity and soil properties. As shrubs have longer root systems and deeper roots than herbaceous plants, the deep soil water content played an important role in the development of the shrub layer, and soil water content was mostly regulated by precipitation as a way to promote community development, whereas the available phosphorus and potassium promote the formation and growth of shrub root systems (Li et al., 2009). In summary, the process of plant community growth and succession in arid desert ecosystems is a process of plant adaptation to soil nutrient changes in their habitats, as well as the interaction of different soil environmental factors (**Figure 8**).

## 5 Conclusion

After fencing restoration, the *Reaumuria songorica*–*Nitraria tangutorum* community in Hexi Corridor, northwest China, was dominated by Zygophyllaceae, the shrub layer was dominated by *Reaumuria songorica*, and the community evolved from simple to complex. The species diversity index of the community increased, then decreased, then increased again as the duration of fencing restoration increased. This change in community diversity was mainly influenced by the role of shrub conservation and soil physicochemical properties. As the shrub layer density increased, its conservation effect promoted an increase of herbaceous plant diversity and density, albeit at a lower rate. These results indicate that vegetation recovery in desert ecosystems is very slow and may take longer than that in less extreme environments.

## Data availability statement

The original contributions presented in the study are included in the article/supplementary material. Further inquiries can be directed to the corresponding author.

## Author contributions

YaZ and GW wrote the manuscript. GW and QG reviewed and edited the manuscript. YuZ, JL and MG provided assistance for data analysis. All authors contributed to the article and approved the submitted version.
